# Reveals of candidate active ingredients in Justicia and its anti-thrombotic action of mechanism based on network pharmacology approach and experimental validation

**DOI:** 10.1038/s41598-021-96683-z

**Published:** 2021-08-25

**Authors:** Zongchao Hong, Ting Zhang, Ying Zhang, Zhoutao Xie, Yi Lu, Yunfeng Yao, Yanfang Yang, Hezhen Wu, Bo Liu

**Affiliations:** 1 Faculty of Pharmacy, Hubei University of Chinese Medicine, Wuhan, 430065 China; 2 Affiliated Hospital of Hubei University of Chinese Medicine, Hubei Hospital of Traditional Chinese Medicine, Wuhan, China; 3 Key Laboratory of Traditional Chinese Medicine Resources and Chemistry of Hubei Province, Wuhan, China; 4 Collaborative Innovation Center of Traditional Chinese Medicine of New Products for Geriatrics Hubei Province, Wuhan, China

**Keywords:** Computational biology and bioinformatics, Drug discovery, Molecular biology, Drug development

## Abstract

Thrombotic diseases seriously threaten human life. Justicia, as a common Chinese medicine, is usually used for anti-inflammatory treatment, and further studies have found that it has an inhibitory effect on platelet aggregation. Therefore, it can be inferred that Justicia can be used as a therapeutic drug for thrombosis. This work aims to reveal the pharmacological mechanism of the anti-thrombotic effect of Justicia through network pharmacology combined with wet experimental verification. During the analysis, 461 compound targets were predicted from various databases and 881 thrombus-related targets were collected. Then, herb-compound-target network and protein–protein interaction network of disease and prediction targets were constructed and cluster analysis was applied to further explore the connection between the targets. In addition, Gene Ontology (GO) and pathway (KEGG) enrichment were used to further determine the association between target proteins and diseases. Finally, the expression of hub target proteins of the core component and the anti-thrombotic effect of Justicia’s core compounds were verified by experiments. In conclusion, the core bioactive components, especially justicidin D, can reduce thrombosis by regulating F2, MMP9, CXCL12, MET, RAC1, PDE5A, and ABCB1. The combination of network pharmacology and the experimental research strategies proposed in this paper provides a comprehensive method for systematically exploring the therapeutic mechanism of multi-component medicine.

## Introduction

The advancement of medical technology and the effective control of fatal diseases have greatly increased the average life span of human beings. Thrombotic diseases, including pulmonary embolism, venous embolism, arterial embolism, cerebral embolism, etc., have seriously threatened human life. Sadly, the global public awareness of thrombotic diseases, especially pulmonary embolism and deep vein thrombosis, is not high. What needs more vigilance is that thromboembolic conditions have been estimated to account for 1 in 4 deaths worldwide in 2010^1^. As a threatening disease, thrombosis can be accompanied by cancer^2^, obesity, and inflammatory bowel disease^3^. Clinical data of patients with coronavirus disease (COVID-19) in 2019 also reported high venous thromboembolism and diffuse intravascular coagulation, and thrombotic complications even became a sign of severe COVID-19^4,5^.

The etiology and pathogenesis of thrombotic diseases are very complex and have not yet been fully clarified. However, recent studies have shown that the occurrence and development of thrombotic diseases are mainly related to five reasons: (1) Vascular endothelial damage^6^; (2) Increased platelet count^7^; (3) Increased blood coagulation^8^; (4) Decreased anti-coagulant activity^9^; (5) Decreased fibrinolytic activity^10^. At present, the treatments for thrombotic diseases mainly includes anti-coagulation therapy, anti-platelet drug therapy, thrombolytic therapy, interventional therapy, and surgery^11^. However, currently commercially available anti-thrombotic drugs do not seem to be fully applicable to all patients. Therefore, the development and use of new anti-thrombotic drugs is inevitable.

Natural products, as a treasury for the development and discovery of new medicines, are worthy of our in-depth exploration. The discovery of anti-thrombotic active ingredients from natural products can effectively enrich the drug bank for the treatment of thrombosis. Justicia (*Justicia procumbens* (L.) Ness), a resource-rich botanical, has been found to have a variety of pharmacological activities including anti-inflammatory, liver protection, anti-oxidation, and anemia treatment^12–14^. Even more surprising is that we accidentally discovered its anti-platelet aggregation ability^15^. A further conjecture is that the Justicia has a satisfactory anti-thrombotic effect or can assist the treatment of thrombotic diseases. However, the research evidence on the anti-thrombotic effect of Justicia is scarce, so that we do not know its anti-thrombotic mechanism.

The network pharmacology proposed by Hopkins University in 2008, is a big data integration method based on a large number of database resources and statistical algorithms. It is used to explore the synergy of multiple components, multiple targets, and multiple mechanisms for the treatment of diseases. From this point of view, the research strategy of network pharmacology is actually consistent with the theory of Chinese medicine. Network pharmacology is a new field based on the development of "ingredient-target-disease" interaction network multidisciplinary integration theory, which provides a new way for modern medicine to explain the responsibility system of Chinese medicine, which also provides a novel strategy for our research purposes^16^. Traditional Chinese Medicine (TCM) treats individuals or patients as systems in different states and has accumulated numerous herbal prescriptions. The overall concept of TCM has a lot in common with the core ideas of emerging network pharmacology and network biology. Based on the existing network pharmacology technology, it is necessary to establish a new TCM network pharmacology method to predict the target spectrum of herbal compounds and pharmacological effects, reveal the drug-gene-disease co-module association, screen synergistic multiple compounds from herbal formulas in a high-throughput manner, and explain the combination rules and network regulation mode of action. Furthermore, the combination of computing, experimentation, and clinical practice is a promising strategy and direction for the future research of TCM network pharmacology^17,18^.

In this work, we first used network pharmacology to analyze the active components and signaling pathways of the anti-thrombotic effect of Justicia, and then verified the results using component analysis, in vivo and in vitro experiments, and GeneChip Human Gene Array.

## Materials and methods

### Chemical database collection

All components of Justicia were obtained from A high-throughput experiment- and reference-guided database of traditional Chinese medicine (HERB, http://herb.ac.cn) and The PubChem Project (https://pubchem.ncbi.nlm.nih.gov)^19,20^. HERB, a high-throughput experiment and reference guide database for traditional Chinese medicine, associates 12,933 targets and 28,212 diseases with 7263 herbs and 49,258 components, and provides six paired relationships in HERB, which will greatly support the modernization of traditional Chinese medicine and guide reasonable modern drug discovery.

### Compounds target fishing

As described by Hsin-Yi Lin et al.^21^, we obtained the SMILES format of Justicia’s ingredients from the PubChem database and fished out the potential targets of compounds from HitPick (http://mips.helmholtz-muenchen.de/hitpick/), similarity ensemble approach (SEA, http://sea.bkslab.org/), Target Hunter of Small Molecule (https://www.cbligand.org/TargetHunter/) database. Then, we integrated the targets that were caught and removed duplicates (ensure the uniqueness of each target).

### Targets of thrombus

The thrombus targets were gathered from the GeneCards database (https://www.genecards.org/)^22^, which provides information about disease targets. The keywords “thrombus” were used, and a total of 881 targets were gathered.

### Obtaining protein–protein interaction data

The targets to be analyzed were imported into the STRING database (https://string-db.org/) to obtain the protein–protein interaction (PPI) relationship, and the obtained PPI data was exported for network visualization.

### Network construction and analysis

All networks were constructed in Cytoscape 3.8.1 software (https://cytoscape.org/download.html), and the visualization network is composed of nodes and edges. Nodes represent components, targets, and herbs.

To study further into the network, MCODE and Cytohubba plugin were introduced to generate clusters and identify hub nodes. The MCODE plugin can determine the main center of the network and generate clusters of interconnected sub-clusters by setting K-cores. Increasing the value of K-core will generate fewer clusters and exclude smaller clusters. Moreover, we can find the node with the highest score, called SEEDs, which may have the opportunity to become the key target of this cluster. Hub genes are usually representative genes. Cytohubba is a plugin used by Cytoscape software to identify hub nodes. The larger the score of the analysis result, the more critical the node and the darker the color displayed.

### Gene ontology and KEGG enrichment analysis

Gene Ontology (GO) enrichment analysis is to show the biological process (BP), cellular component (CC), and molecular function (MF) that genes affect together. Kyoto Encyclopedia of Genes and Genomes (KEGG) signaling pathway enrichment analysis can discover the importance of signaling pathways involved in genes. Metascape (http://metascape.org/) is a powerful gene function annotation analysis tool. It integrates multiple authoritative data resources such as GO, KEGG, UniProt, and DrugBank, so that it can not only complete pathway enrichment and biological process annotation, but also do gene-related protein network analysis and predictive analysis of transcription regulation involved^23^. Therefore, we employed metascape for the enrichment analysis of this study.

### Reagents and materials

DMSO (#30072418), NaCl (#10019318), KH_2_PO4 (#10017618), Na_2_HPO4·12H_2_O (#2006062), and KCl (#130412) are all analytical pure, provided by Sinopharm Chemical Reagent Co., Ltd. (Shanghai, China); ADP (#11607920, Sigma, USA), Aspirin (#A2093, Sigma, USA), TRIzol (#15596026, Thermo Fisher Scientific, USA); water was prepared by Millipore Milli-Q; Justicidin D was purified by the laboratory (purity > 95%, the results of ^1^H-NMR identification are shown in Figure S1 in the support material). Human–machine platelet collection is provided by Wuhan Blood Center (blood for scientific research, approved by Hubei Provincial Blood Management Center, Wuhan, China). RNA extraction kit (QIAGEN, Germany), Pico Reagent Kit and GeneChip Human Gene 1.0 ST Array (Affymetrix, USA). GeneChip scanner (30007G, Affymetrix, USA). Rat tail tendon collagen type I (#C8062) was purchased from solarbio (Beijing, China). Adrenalin hydrochloride was purchased from Tianjin Jinyao Pharmaceutical Co., Ltd. (Tianjing, China).

PBS: 8 g NaCl, 0.2 g KCl, 1.44 g Na_2_HPO4, and 0.24 gKH_2_PO4, dissolved in 900 ml ultra pure water, adjust the pH to 7.4 with hydrochloric acid, add water to 1000 ml, and autoclave for 20 min.

Justicidin D was prepared into 6.8 mg/ml stock solution in DMSO and diluted to 0.34 mg/ml with PBS. ADP could induce platelet aggregation, it was prepared as a 0.12 mg/ml solution with PBS when used. Aspirin was formulated with PBS as a stock solution at a concentration of 10 mM.

### Transcriptional sequencing

The RNA expression of platelets was analyzed in blank group (treatment with 5% DMSO 45 μl and PBS 15 μl) and justicidin D group (treatment with 15 μl of ADP solution and 45 μl of justicidin D solution). Each group was reacted in a water bath at 37 °C for 5 min, and then centrifuged at 3000 rpm for 3 min. After repeated washing and centrifugation with PBS for 2 times, 2 ml of TRIzol was added for total RNA extraction.

Follow the instructions of the RNA extraction kit to extract platelet RNA. After quality testing, high-quality samples (OD260/280 = 1.8–2.2, OD260/230 ≥ 2.0, RIN ≥ 6.5, 28S:18S ≥ 1.0) were used to construct sequencing libraries. Then, cDNA reverse transcription synthesis was performed according to the kit instructions. Briefly, double-stranded cDNA was synthesized using the superscript double-stranded cDNA synthesis kit (Pico Reagent Kit) and random hexamer primers. According to the Illumina library construction program, the synthesized cDNA was repaired, phosphorylated, and added 'A' base. The size of the library was 200–300 bp, and it was screened with 2% low-range ultra-agarose, followed by PCR amplification. After TBS380 quantification, the paired-end RNA seq sequencing library was used to hybridize with the GeneChip Human Gene 1.0 ST Array chip. Finally, get the scanned data.

### Differential expression analysis

To identify differential expression genes (DEGs) between two different group samples, The R statistical package software EdgeR (http://www.bioconductor.org/packages/2.12/bioc/html/edgeR.html) was used to assess differential expression. After corrected for multiple hypothesis testing, |Fold change|> 1, and *p* < 0.05 was the threshold for screening DEGs. Hiplot (https://hiplot.com.cn/basic) and bioinformatics (http://www.bioinformatics.com.cn/) was used for visualization of results.

### Anti-platelet aggregation

Turbidimetric platelet aggregometry (TPA) is the most widely used and classical method for clinical determination of platelet aggregation rate^24^. Take machine-collected platelets, centrifuge at 800 rpm for 8 min, and suck the upper layer of platelets for the experiment. Grouped into: blank group, ADP group, justicidin D group, and aspirin group (Positive control). The blank group was treated with 5% DMSO solution (diluted with PBS), and the ADP group was the model group. In addition to ADP treatment, justicidin D group and aspirin group also gave justicidin D and aspirin treatment, respectively.

Percentage of platelet aggregation: Take 260 μl of platelets (3 × 10^8^·ml^−1^) and store them in a reaction cup preheated at 37 °C with a magnetic rod. Add 30 μl of the drug solution and incubate for 25 min. While magnetic stirring, add ADP inducer 10 μl. The platelet-removed plasma reaction tube set in parallel was used for zero adjustment, and the platelet-rich reaction tube was adjusted to 100%, and the light transmittance of each tube was measured to record the maximum platelet aggregation rate within 5 min. In the blank group, 30 μl of 5% DMSO solution was used to replace the drug solution to perform the same operation. Platelet aggregation inhibition rate (%) = (1–(maximum aggregation percentage of experimental group / maximum aggregation percentage of blank group)) × 100% (n = 3).

### Animals

40 male Balb/c mice (SPF grade) weighing 18–22 g were provided by the Experimental Animal Center of China Three Gorges University. Randomly divided into 4 groups (blank group, model group, aspirin group, justicidin D group), 10 animals in each group, reared in a standard environment, environment temperature 22 °C, relative humidity 30–50%, 12 h light–dark cycle. Mice in each group were intragastrically administered with the corresponding concentration of medicinal solution for 7 consecutive days. The mice were fasted 12 h before the last administration, and the models were made 1 h after the last administration. Rat tail tendon collagen type I (500 μg/ml) and adrenalin hydrochloride (15 μg/ml) mixture (v:v = 1:1) were injected into the tail vein of the model group and the administration group, and the blank group was injected into the tail vein with equal volume of saline. The volume of intragastric administration is 0.2 ml/10 g body weight, and the volume of tail vein injection is 0.1 ml/10 g body weight. Observe the physiological behavior of the mice within 30 min after modeling, and calculate the protection rate. Take lung slices for HE staining to observe thrombosis. Protection rate% = (number of surviving mice in the drug treatment group/number of surviving mice in the blank group) × 100%.

Animal welfare and experimental procedures strictly follow the guidelines of the Animal Research Committee of Hubei University of Chinese Medicine, comply with the European Community guidelines (EEC Directive of 1986; 86/609/EEC), and carried out in compliance with the ARRIVE guidelines.

### Statistical analysis

Student's t-test was used to compare gene expression between different groups. The statistical value p < 0.05 indicates that the difference is statistically significant. Graphpad Prism 8.0 was also used for statistical analysis.

### Statement

Animal experimental research has been approved by the Animal Research Committee of Hubei University of Chinese Medicine. Animal welfare and experimental procedures strictly follow the guidelines of the Animal Research Committee of Hubei University of Chinese Medicine, comply with the European Community guidelines (EEC Directive of 1986; 86/609/EEC), and carried out in compliance with the ARRIVE guidelines.

### Informed consent

The informed consent of the blood contributor was obtained, and protocols were approved by the ethics committee of Hubei Provincial Hospital of Traditional Chinese Medicine. The research process followed the Declaration of Helsinki made by the World Medical Association (WMA).

## Results

### Components of Justicia and targets

We collected 39 Justicia candidates from HERB database for further analysis. At the same time, we predicted the possible targets of each compound and obtained a total of 460 single targets (Supplementary Table S1). Notably, the same compound has multiple targets, and different compounds may also act on the same target, which conforms to the characteristics of multi-component and multi-target of botanicals. 881 genes related to thrombus were collected from the GeneCards database, and we used the top 400 genes for the next analysis.

### Topology network construction

**Figure 1:**
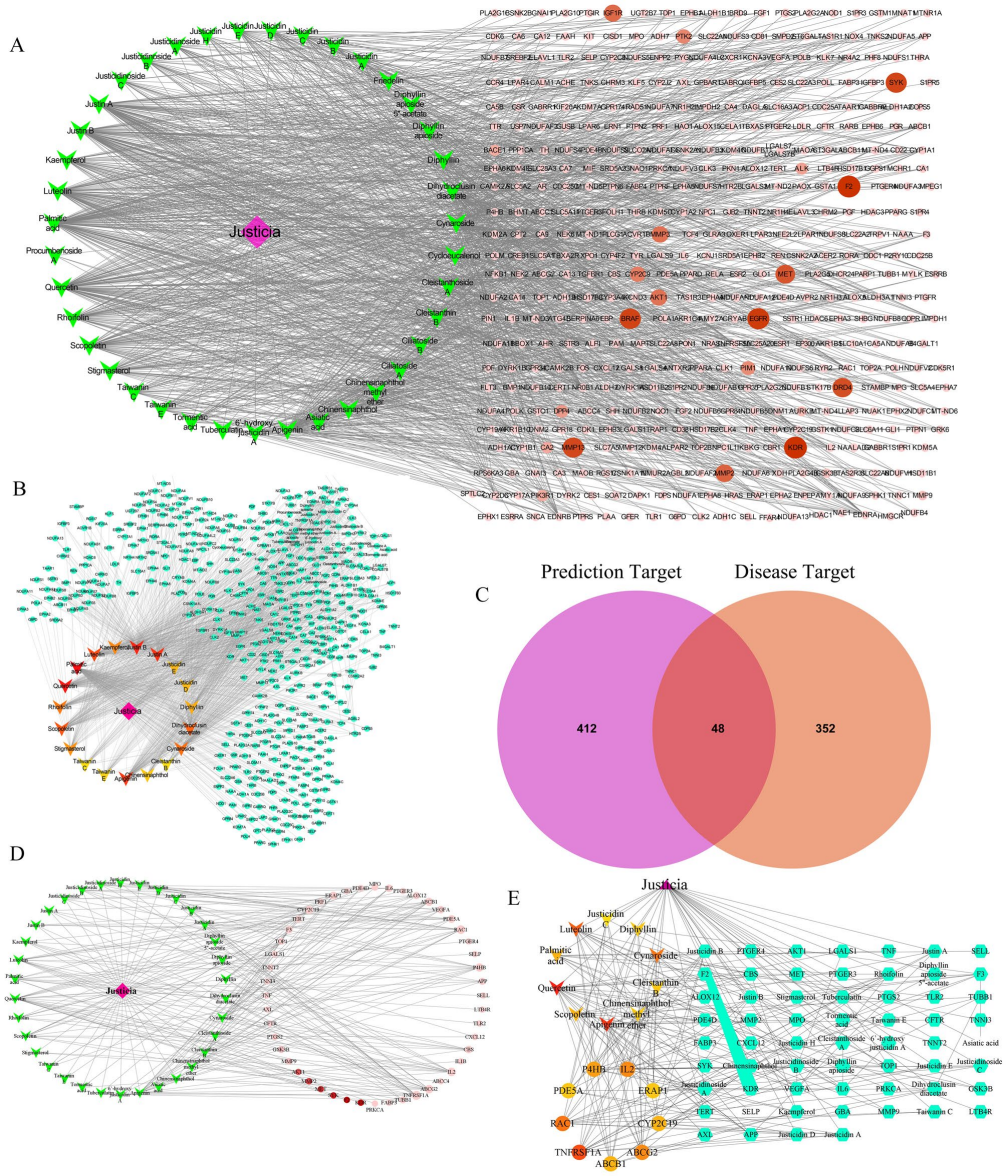
Constructed network related to Herb-compounds-targets. (**A**) Herb-compounds-predicted targets network. (**B**) The core components in the Herb-compound-predicted target network calculated by Cytohubba (top 20). (**C**) The intersection of justicia's compound predicted targets and thrombus-related targets, with 48 common targets. (**D**) Herb-compounds-common targets network. (**E**) The 11 core components and 9 hub targets in the herb-compounds-common targets network calculated by Cytohubba. The purple square represents the botanical justicia, the three-pointed star represents the compound, and the circle represents the target. For the targets in (**A**,**D**), the darker the circle, the larger the degree. The light yellow to dark orange gradient in (**B**,**E**) indicates that the corresponding node is more important.

39 compounds were collected in Justicia, and 460 possible targets were predicted, so we used Cytoscape software to construct the herb-compound-target topology network (Fig. 1A). The network contains a total of 500 nodes (one herb, 39 compounds, 460 targets), 1402 edges. Among these components, palmitic acid, quercetin, justin B, and justin A have the highest number of targets. Correspondingly, EDNRB, EDNRA, and NROB1 are the common targets of more than half of the compounds. In depth, we use the cytohubba plug-in to analyze the topological network and get the core compounds and the corresponding targets (Fig. 1B), and the top 20 nodes are shown in Table 1. Undoubtedly, palmitic acid, quercetin, Justin B, and Justin A are at the top of the list. What is more surprising is that justicidin E and justicidin D, the characteristic ingredients of Justicia, are among the top 15 compounds.

**Table 1:** Top 20 nodes in Merged Network constructed by Herb-Compounds-Prediction targets.

Rank	Name	Score	PubChem CID
1	Palmitic acid	117	985
2	Quercetin	98	5,280,343
3	Justin B	89	10,672,178
4	Justin A	84	10,551,264
5	Apigenin	82	5,280,443
6	Luteolin	80	5,280,445
7	Scopoletin	70	5,280,460
8	Dihydroclusin diacetate	68	21,600,064
9	Cynaroside	64	5,280,637
10	Rhoifolin	52	5,282,150
11	Kaempferol	48	5,280,863
12	Stigmasterol	45	5,280,794
13	Justicia	39	N/A
14	Diphyllin	33	100,492
15	Justicidin E	32	363,128
16	Justicidin D	32	5,318,737
17	Chinensinaphthol	30	332,529
18	Taiwanin E	29	493,164
19	Taiwanin C	28	363,127
20	Cleistanthin B	28	119,458

Under the premise of analyzing the anti-thrombotic effect of Justicia, we included 400 targets related to thrombus into the analysis process. Among these 400 targets, 48 were predicted by 34 Justicia compounds (It can be called common targets, shown in Supplementary Table S2, Fig. 1C). Similarly, we constructed a topological network of 34 compounds and 48 shared targets (Fig. 1D). There were 224 edges in this network, which means that there were 224 connections between 34 compounds and 48 targets. Not surprisingly, under Cytohubba's analysis, justicidin D remains one of the core nodes. In addition, nine targets, TNFRSF1A, RAC1, ABCG2, IL2, P4HB, CYP2C19, ABCB1, ERAP1, and PDE5A, also appeared in the top 20 core nodes (Fig. 1E, Table 2). In-depth understanding is that these nine targets may play a vital role in the anti-thrombotic effect of Justicia.

**Table 2:** Top 20 nodes in the network constructed by Herb-Compounds-Common targets.

Rank	Name	Score
1	Justicia	34
2	Quercetin	19
3	Apigenin	18
4	TNFRSF1A	15
5	Luteolin	14
6	Cynaroside	13
7	RAC1	13
8	ABCG2	12
9	IL2	12
10	P4HB	11
11	Scopoletin	10
12	Palmitic acid	10
13	CYP2C19	10
14	ABCB1	10
15	Chinensinaphthol methyl ether	8
16	Cleistanthin B	8
17	Diphyllin	8
18	ERAP1	8
19	PDE5A	8
20	Justicidin D	7

### Prediction targets PPI network construction

**Figure 2:**
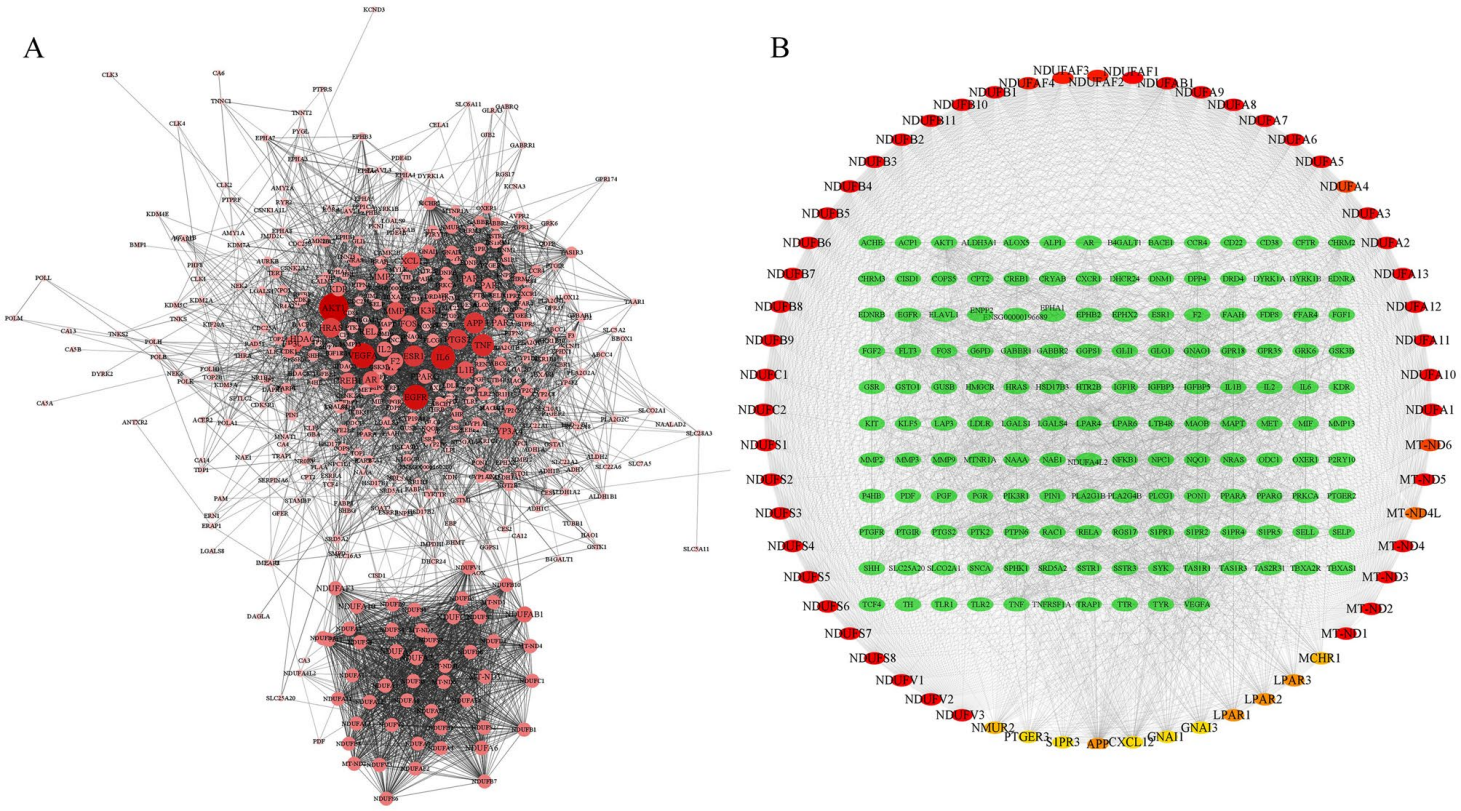
Network analysis on compound predicted targets. (**A**) The protein–protein interaction (PPI) network of 460 targets predicted by compounds. The closer, redder and the larger the nodes are, the higher the degree of freedom they have. (**B**) Important nodes in PPI network (top 60, calculated by cytohubba), the darker the nodes, the higher their importance.

The compounds in Justicia predicted 460 targets. The symbols of these targets were input into STRING database, species were limited to “Homo sapiens”. Then, it was imported into Cytoscape3.8.1 to construct the network (Fig. 2A). After PPI was acquired, cytohubba calculated the top 60 target networks (Fig. 2B), among which MT-ND5, MT-ND2, MT-ND4, NDUFS2, and NDUFA8 ranked in the top five.

### Clusters of prediction target PPI network

**Figure 3:**
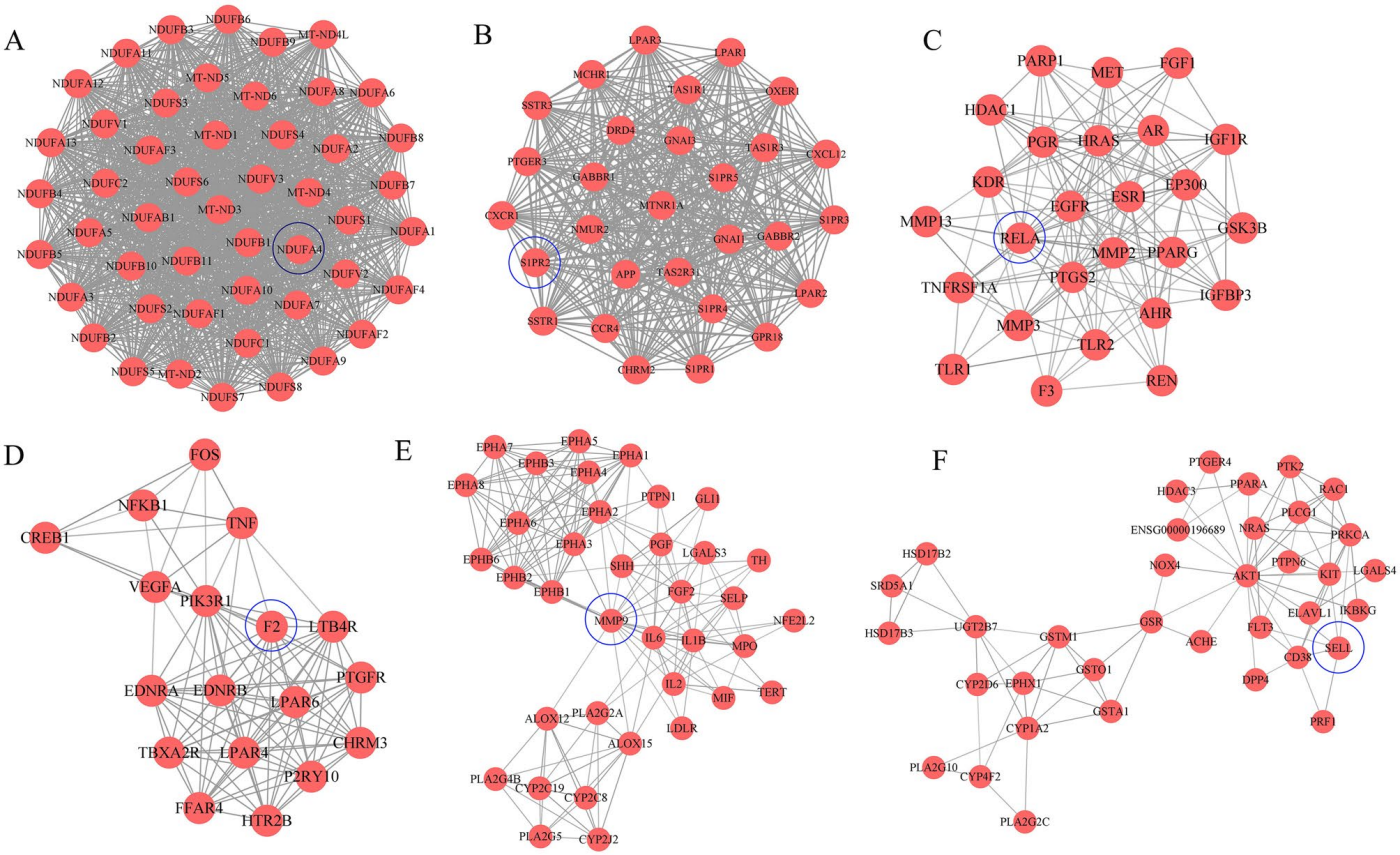
Clusters of compound predicted targets PPI network. (**A**–**F**) are clusters we found in the predicted targets PPI network which stands for cluster 1 to 6, respectively. The seed node of each clusters is marked with blue circles.

MCODE plug-in is used for cluster analysis. Six clusters were obtained after conducting clustering analysis for the prediction target network, and each cluster has seed nodes (marked by a blue circles). The details are shown in Fig. 3 and Table 3.

**Table 3:** Clusters of the predicted target PPI network.

Cluster	Score	Nodes	Edges	Gene IDs
1	48.83	49	1172	NDUFS2, NDUFA8, NDUFA3, NDUFA1, NDUFA11, NDUFA13, NDUFB2, NDUFC1, NDUFAF4, MT-ND6, NDUFC2, NDUFA4, NDUFB1, NDUFV2, NDUFV1, NDUFS8, NDUFA7, NDUFB8, NDUFAF2, NDUFB11, NDUFS6, NDUFA9, NDUFS3, NDUFAF1, NDUFS7, NDUFB7, MT-ND1, NDUFS5, NDUFB4, NDUFS1, NDUFB10, MT-ND3, NDUFAF3, NDUFA5, NDUFV3, NDUFS4, NDUFA10, NDUFB5, NDUFB3, NDUFA6, NDUFB6, NDUFA12, NDUFAB1, NDUFA2, NDUFB9, MT-ND5, MT-ND2, MT-ND4, MT-ND4L
2	29.00	29	406	GNAI1, S1PR2, CCR4, S1PR5, OXER1, CHRM2, CXCR1, S1PR4, LPAR2, LPAR3, APPG, ABBR1, SSTR3, MTNR1A, CXCL12, TAS1R3, TAS1R1, DRD4, TAS2R31, S1PR1, GPR18, S1PR3, LPAR1, GABBR2, GNAI3, SSTR1, NMUR2, MCHR1, PTGER3
3	12.24	26	153	GSK3B, MMP3, HRAS, IGF1R, ESR1, MMP13, TLR2, FGF1, PTGS2, MMP2, TNFRSF1A, IGFBP3, EGFR, KDR, EP300, AHR, PGR, HDAC1, PPARG, REN, MET, PARP1, RELA, TLR1, AR, F3
4	11.65	18	99	FOS, P2RY10, F2, PIK3R1, NFKB1, FFAR4, TBXA2R, LPAR4, EDNRA, LPAR6, EDNRB, PTGFR, VEGFA, LTB4R, TNF, HTR2B, CHRM3, CREB1
5	9.44	37	170	EPHB6, EPHB3, NFE2L2, LDLR, TERT, CYP2J2, EPHA2, ALOX15, PTPN1, PLA2G2A, EPHB1, PLA2G4B, EPHB2, PLA2G5, EPHA1, SELP, EPHA8, IL6, ALOX12, EPHA5, TH, CYP2C19, CYP2C8, PGF, IL1B, MIF, MPO, EPHA4, FGF2, EPHA3, EPHA6, EPHA7, LGALS3, IL2, SHH, MMP9, GLI1
6	5.03	36	88	PRKCA, PTK2, DPP4, GSR, GSTA1, GSTO1, LGALS4, HDAC3, PTGER4, PLA2G10, AKT1, PLA2G2C, CYP4F2, NOX4, HSD17B3, IKBKG, ENSG00000196689, PLCG1, FLT3, SELL, CYP2D6, HSD17B2, PTPN6, CYP1A2, ELAVL1, CD38, GSTM1, UGT2B7, KIT, ACHE, PRF1, SRD5A1, NRAS, PPARA, EPHX1, RAC1

Cluster 1 contains 49 nodes and 1172 edges with a score of 48.83. The seed node of cluster 1 is NDUFA4, which is a cytochrome c oxidase subunit and has been confirmed that its mutation can cause human diseases, especially nervous system diseases, and is associated with immune response^25,26^.

Cluster 2 contains 29 nodes and 406 edges, with a score of 29.0. The seed node is S1PR2, which plays a vital role in atherosclerosis and can be used as a novel therapeutic target for atherosclerosis. In addition, S1PR2 has been proven to enhance endothelial barrier function in vivo and in vitro, and participate in platelet aggregation^27,28^.

Cluster 3 contains 18 nodes and 99 edges, with a score of 12.24. The seed node is RELA, which mediates the reduction of atherosclerotic plaque in the aortic valve, and targeting RELA can inhibit vascular endothelial dysfunction^29,30^.

Cluster 4 contains 26 nodes and 153 edges, with a score of 11.65. The seed node is F2 (Prothrombin), which functions in blood coagulation, and an unexplainable novel F2 gene mutation for thrombosis was discovered in a Dutch white family^31^.

Cluster 5 contains 37 nodes and 170 edges, with a score of 9.44. The seed node is MMP9, and its protein content and enzyme activity in platelets of myocardial infarction are significantly increased. Furthermore, the overexpression of MMP9 can mediate the pro-angiogenic function in tumors^32,33^.

Cluster 6 contains 36 nodes and 88 edges, with a score of 5.03. The seed node is SELL, which can be used as an indicator to predict myocardial infarction and mediate the interaction between tumor cells and blood components (including platelets, endothelial cells, and white blood cells)^34,35^.

### Disease PPI network construction

**Figure 4:**
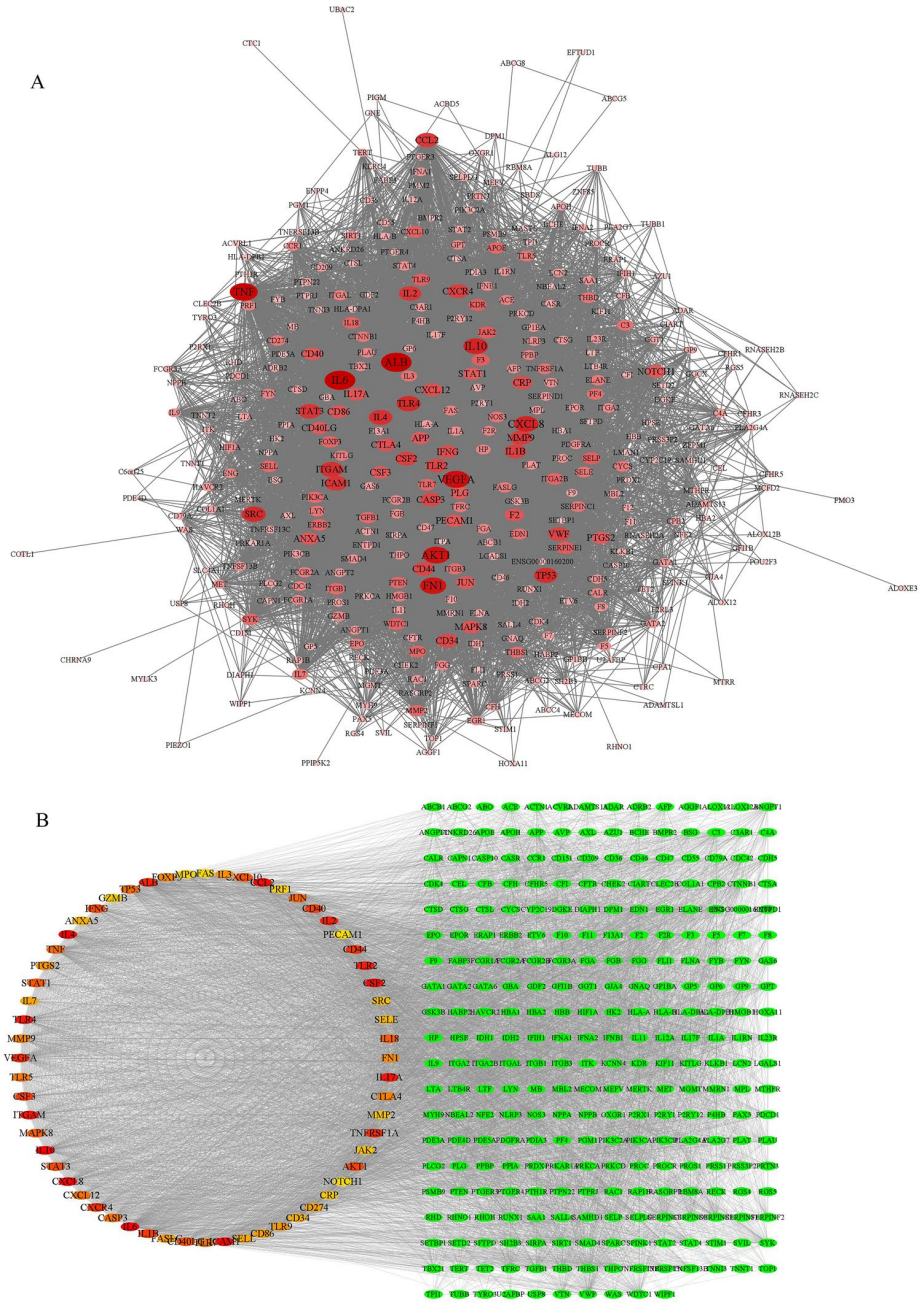
Network analysis on thrombus-related targets. (**A**) The PPI network of the top 400 thrombus-related targets. The closer, redder and the larger the nodes are, the higher the degree of freedom they have. (**B**) Important nodes in PPI network (top 60, calculated by cytohubba), the darker the nodes, the higher their importance.

Based on the results of the database GeneCards, there were a total of 881 candidate targets related to thrombosis. Import these target genes into STRING to get PPI network data. Then, the first 400 targets were imported into Cytoscape 3.8.1 to visualize the network (Fig. 4A). The Maximal Clique Centrality (MCC) algorithm based on Cytohubba calculates the first 60 nodes in the network (Fig. 4B), among which IL6, IL10, CXCL8, IL4, ICAM1 were the most important top 5 nodes.

### Clusters of disease PPI network

**Figure 5:**
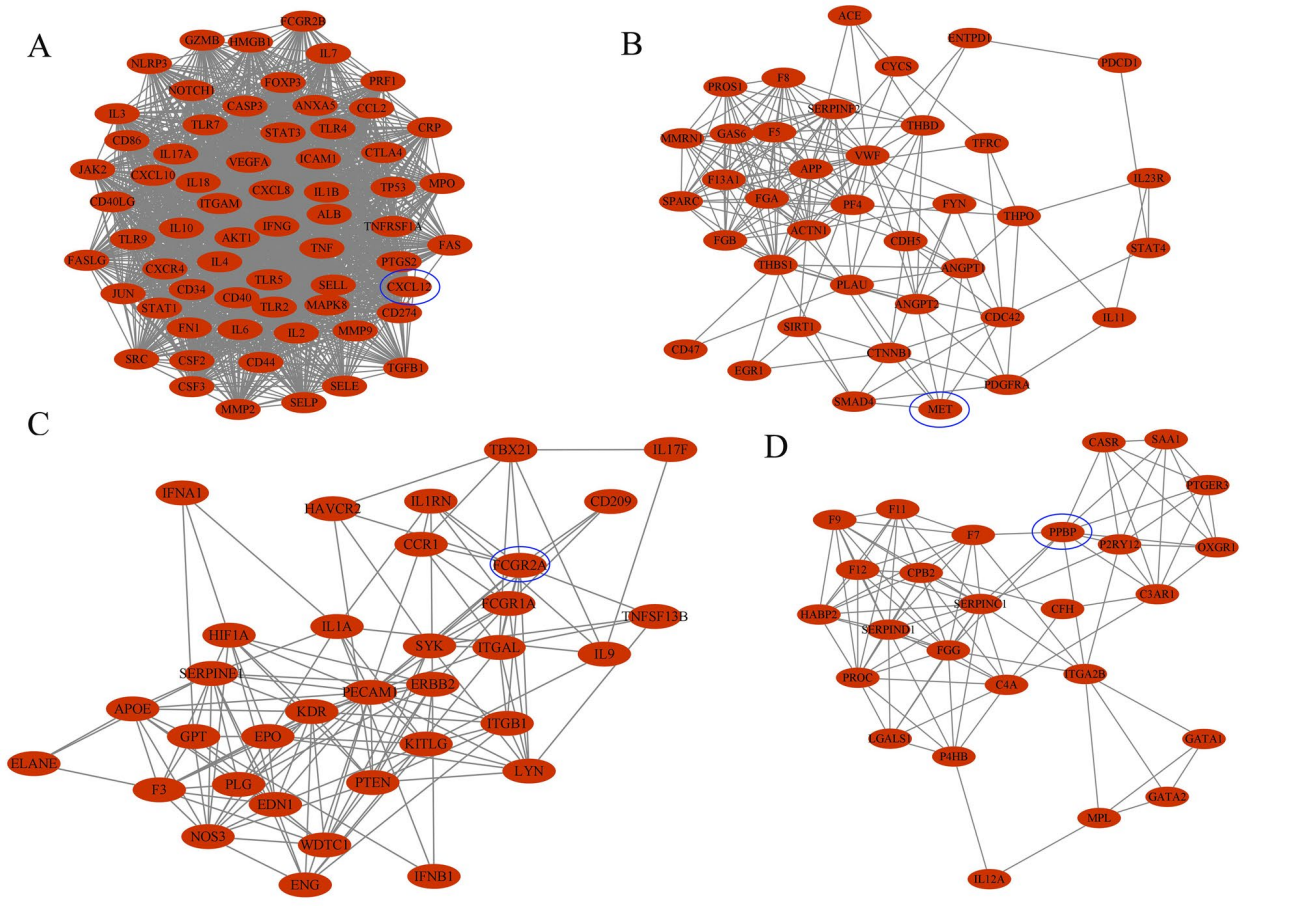
Clusters of thrombus-related targets PPI network. (**A**–**D**) are clusters we found in the thrombus-related targets PPI network which stands for cluster 1 to 4, respectively . The seed node of each clusters is marked with blue circles.

Cluster analysis of thrombus-related gene PPI network, 4 clusters were obtained. The detailed information are shown in Fig. 5 and Table 4. Cluster 1 contains 64 nodes and 1774 edges, with a score of 56.32. The seed node of this cluster is CXCL12, which promotes the uptake of platelet oxidixed low-density lipoprotein (oxLDL) and synergistically enhances the effects of LDL-oxLDL-induced pro-oxidation and pro-thrombosis on platelet function^36^. Cluster 2 contains 38 nodes and 185 edges, with a score of 10.00. The seed node of this cluster is MET (also called HGF), which is an angiogenic factor and a therapeutic target for a variety of solid tumors. The increase in its concentration is also a sign of arterial thrombosis^37,38^. Cluster 3 contains 34 nodes and 148 edges, with a score of 8.97. The seed node of this cluster is FCGR2A (also known as FcγRIIA), which is expressed in human platelets. The expression of FcγRIIA by transgenic technology in lupus mice can trigger major changes in the platelet transcriptome and cause lung and kidney thrombosis^39^. Cluster 4 contains 26 nodes and 104 edges, with a score of 8.16. The seed node of this cluster is PPBP, which is a biomarker of platelet degranulation, and plasma PPBP can activate coagulation^40^.

**Table 4:** Clusters of the disease target PPI network.

Cluster	Score	Nodes	Edges	Gene IDs
1	56.32	64	1774	CRP, FOXP3, TLR4, TLR2, CXCL12, SRC, JUN, FASLG, CD40, CCL2, PTGS2, SELL, MPO, CD40LG, IFNG, TGFB1, IL17A, MMP2, CASP3, CD34, CSF3, FCGR2B, TLR9, ANXA5, GZMB, ALB, STAT1, NOTCH1, FAS, TP53, TNFRSF1A, SELE, STAT3, JAK2, ICAM1, SELP, IL2, NLRP3, CD86, CXCL8, MMP9, CXCR4, HMGB1, ITGAM, TLR5, PRF1, CXCL10, CTLA4, IL10, CSF2, FN1, IL3, IL4, IL18, VEGFA, AKT1, TNF, CD274, CD44, TLR7, IL6, IL1B, IL7, MAPK8
2	10.00	38	185	PROS1, PDGFRA, PLAU, F5, FYN, CTNNB1, MET, GAS6, SERPINF2, ANGPT1, FGA, CD47, FGB, CYCS, ACE, TFRC, PF4, IL23R, THBD, SIRT1, F13A1, THPO, CDC42, F8, THBS1, CDH5, ENTPD1, SPARC, APP, EGR1, STAT4, SMAD4, ANGPT2, ACTN1, VWF, PDCD1, IL11, MMRN1
3	8.97	34	148	HAVCR2, IFNA1, PLG, FCGR2A, TNFSF13B, EPO, PTEN, IL17F, ELANE, GPT, ITGB1, SYK, IL1A, SERPINE1, HIF1A, F3, FCGR1A, LYN, WDTC1, ENG, IL1RN, PECAM1, CD209, KITLG, CCR1, IL9, ERBB2, TBX21, IFNB1, KDR, ITGAL, NOS3, EDN1, APOE
4	8.16	26	104	CPB2, F11, F12, CFH, MPL, SAA1, FGG, P4HB, OXGR1, IL12A, CASR, P2RY12, SERPINC1, PPBP, PTGER3, F7, GATA2, C3AR1, PROC, GATA1, F9, LGALS1, SERPIND1, C4A, HABP2, ITGA2B

### Enrichment analysis

In the network construction stage, 48 genes were found to exist in both thrombosis-related genes and compound prediction genes, and Fig. 1E shows the core 9 of the 48 genes. Therefore, we imported 48 common genes and 9 key genes into the Metascape database for enrichment analysis and selected the top 10 of each category for display.

**Figure 6:**
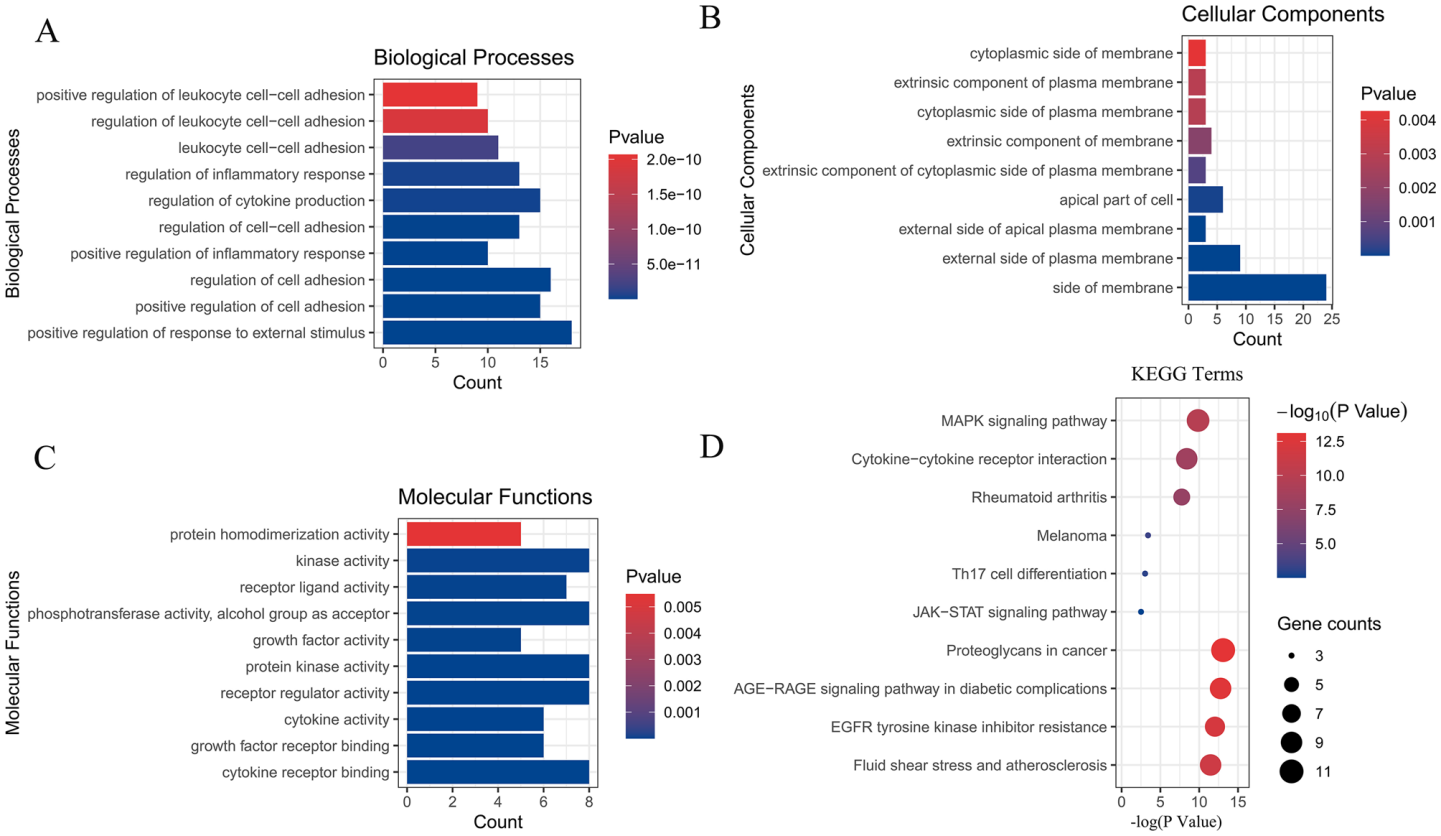
GO and KEGG enrichment analysis of 48 common targets. (**A**–**C**) are biological processes, cellular components, and molecular functions in GO analysis. D is the KEGG enrichment analysis item. All show the top 10.

For the enrichment results of 48 genes, the biological process (BP) is shown in Fig. 6A, including regulation of leukocyte cell–cell adhesion (GO ID: 1903037), regulation of inflammatory response (GO ID: 0050727), regulation of cell adhesion (GO ID: 0030155), and regulation of response to external stimulus (GO ID: 0032103). Cellular component (CC) is shown in Fig. 6B, including side of membrane (GO ID: 0098552), external side of plasma membrane (GO ID: 0009897), apical part of cell (GO ID: 0045177), cytoplasmic side of membrane (GO ID: 0098562), and extrinsic component of membrane (GO ID: 0019898). Molecular function (MF) as shown in Fig. 6C, includes cytokine receptor binding (GO ID: 0005126), cytokine activity (GO ID: 0005125), protein homodimerization activity (GO ID: 0042803), and kinase activity (GO ID: 0016301). In addition, KEGG is shown in Fig. 6D, including MAPK signaling pathway (KEGG ID: hsa04010), Cytokine-cytokine receptor interaction (KEGG ID: hsa04060), Th17 cell differentiation (KEGG ID: hsa04659), and JAK-STAT signaling pathway (KEGG ID: hsa04630). The details of the above enriched entries are described in Supplementary Table S3.

**Figure 7:**
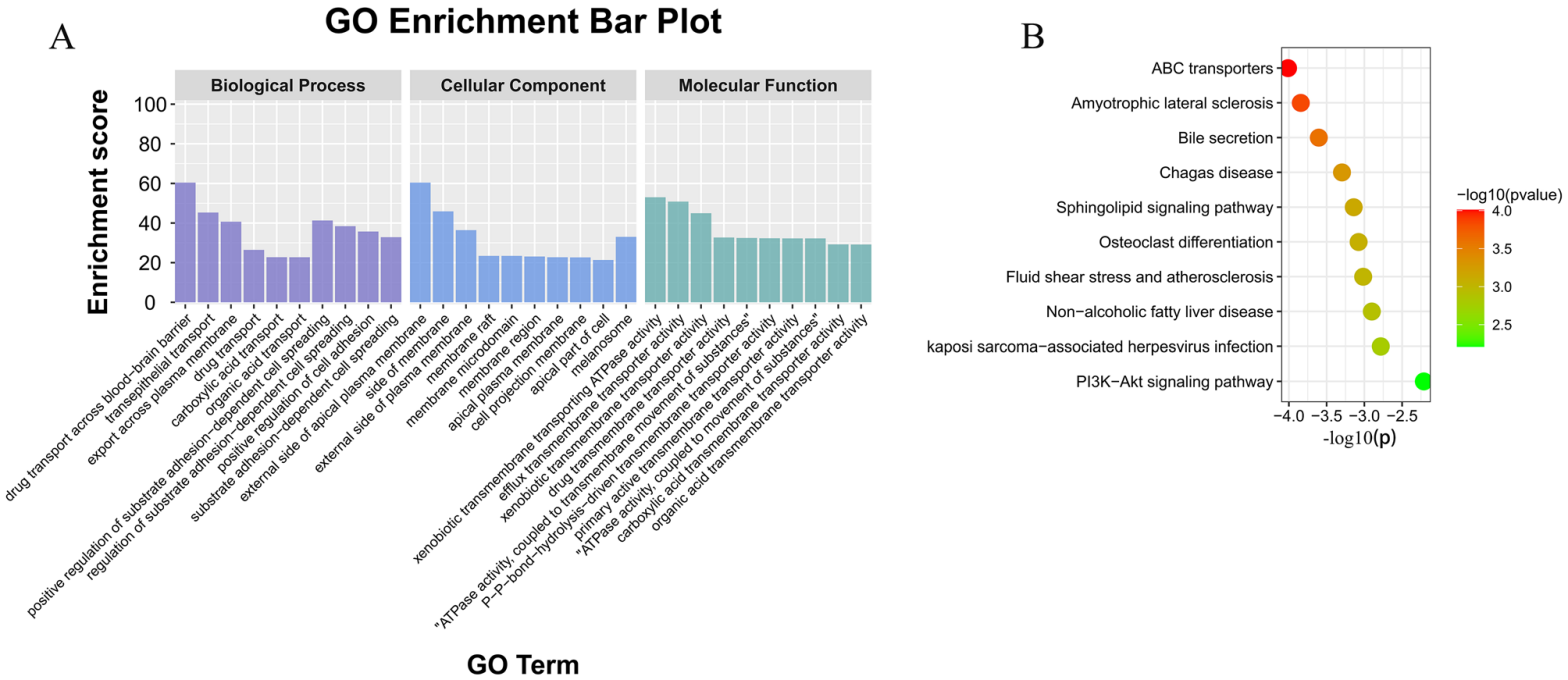
GO and KEGG enrichment analysis of 9 hub targets. A and B represent the first 10 entries enriched by GO and KEGG, respectively.

**Figure 8:**
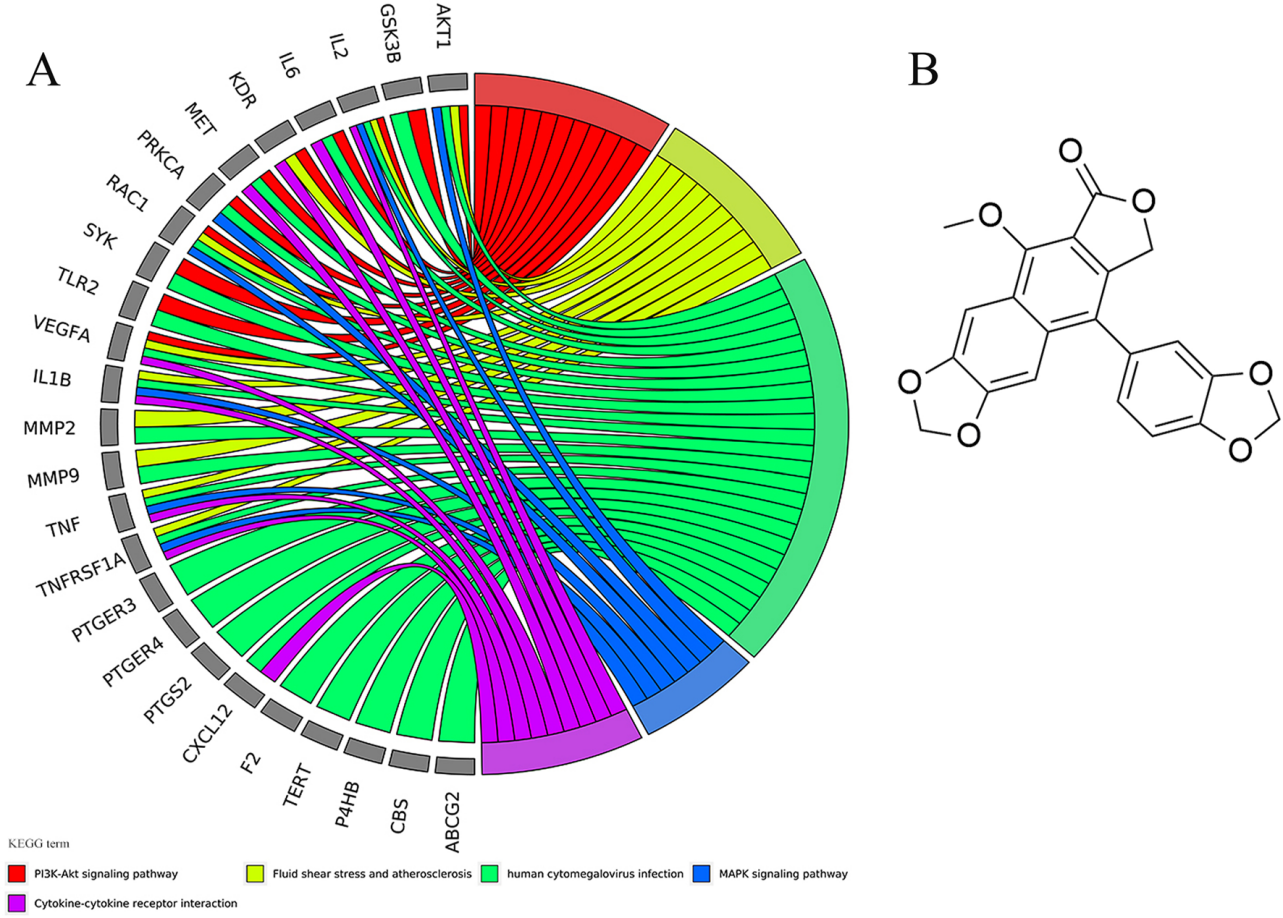
(**A**) 5 overlaps among the top 20 KEGG entries enriched by 48 common targets and 9 hub targets. (**B**) The structure of justicidin D.

For the enrichment results of 9 core genes (described in Supplementary Table S4), the BP, CC, and MF are shown in Fig. 7A. It is not difficult to find that compared with the enrichment results of 48 genes, there are similarities in cell adhesion, drug transport, membrane function, and molecular function. For the KEGG enrichment analysis (Fig. 7B shows the first 10 entries), we were not surprised to find in the two enrichment results that 5 of the first 20 entries overlapped, including PI3K-Akt signaling pathway, fluid shear stress and atherosclerosis, human cytomegalovirus infection, MAPK signaling pathway, and Cytokine-cytokine receptor interaction, these signaling pathways are directly or indirectly related to thrombosis. Interestingly, the seed nodes F2, CXCL12, MMP9, and MET in the disease target and compound prediction target network clusters contribute to the enrichment of these five signaling pathways (Fig. 8A, Table 5). This gives us confidence to determine that the core node analysis for the compounds in Justicia and thrombus-related targets is instructive. Core compounds including justicidin D may regulate the expression of F2, CXCL12, MMP9, and MET.

**Table 5:** Five common pathways associated with candidate targets according to enrichment analysis based on KEGG.

ID	Pathway	Log (P)	Gene IDs
hsa04151	PI3K-Akt signaling pathway	− 10.466	AKT1, GSK3B, IL2, IL6, KDR, MET, PRKCA, RAC1, SYK, TLR2, VEGFA
hsa05418	Fluid shear stress and atherosclerosis	− 11.468	AKT1, IL1B, KDR, MMP2, MMP9, RAC1, TNF, TNFRSF1A, VEGFA
hsa05163	Human cytomegalovirus infection	− 15.868	AKT1, GSK3B, IL1B, IL6, PRKCA, PTGER3, PTGER4, PTGS2, RAC1, CXCL12, TNF, TNFRSF1A, VEGFA, F2, IL2, MET, MMP2, MMP9, TERT, SYK, P4HB, TLR2, CBS, ABCG2
hsa04010	MAPK signaling pathway	− 9.852	AKT1, IL1B, PRKCA, RAC1, TNF, TNFRSF1A
hsa04060	Cytokine-cytokine receptor interaction	− 8.396	IL1B, IL2, IL6, KDR, MET, CXCL12, TNF, TNFRSF1A, VEGFA

### Justicidin D causes differential expression of thrombus-related genes

In view of the core analysis of the compound and target network, justicidin D, as one of the core components, proved to be a unique active component in Justicia. Therefore, we conducted an experimental studies on justicidin D.

**Figure 9:**
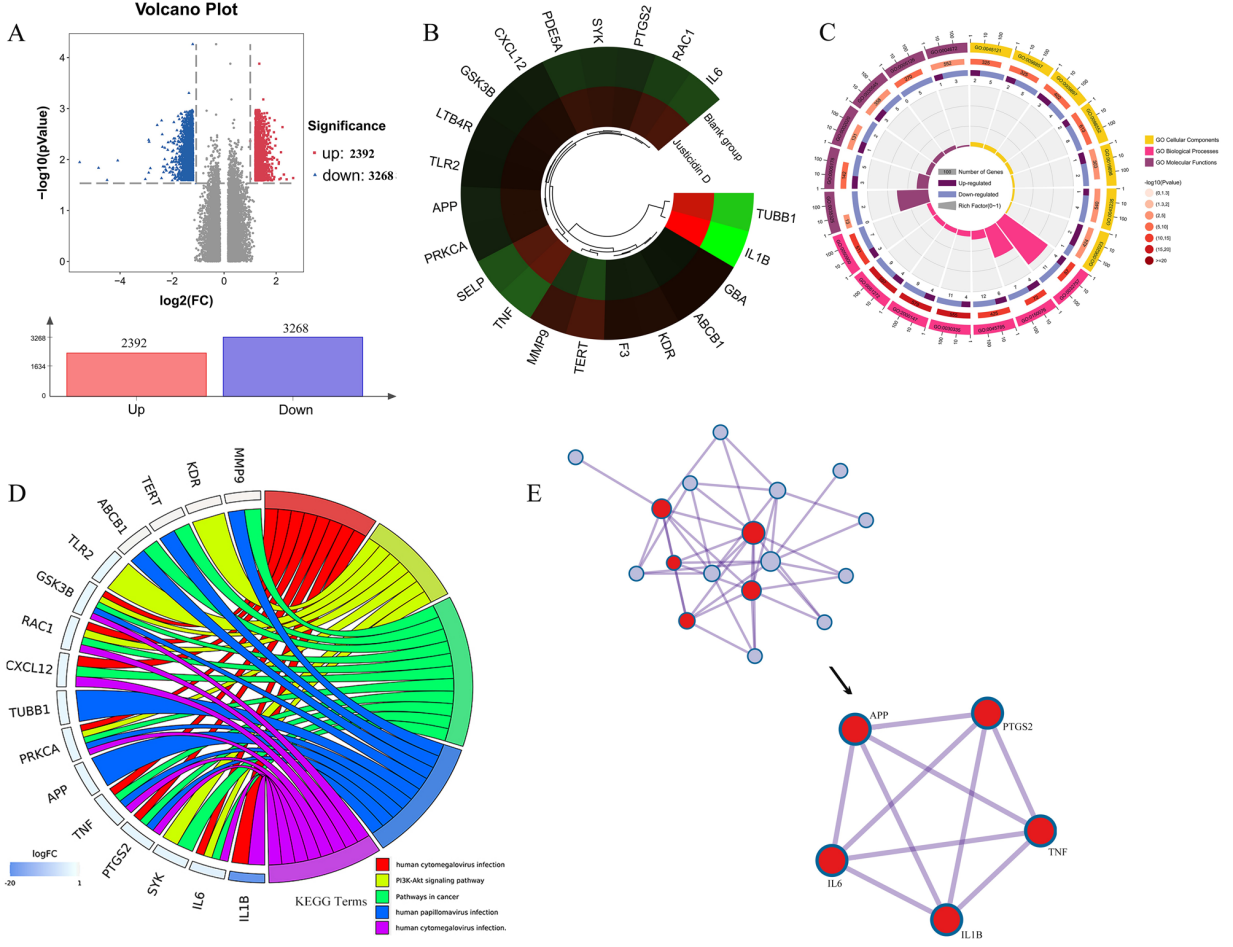
Gene chip detection confirmed that justicidin D can cause the differential expression of 21 genes out of 48 common genes. (**A**) Volcano plot, justicidin D caused high expression of 2392 genes and low expression of 3268 genes. (**B**) Among the 48 common genes in the network analysis, we have determined that 21 genes have significant differential expression. Red is high expression and green is low expression. (**C**) GO enrichment entries for 21 differentially expressed genes. (**D**) KEGG enrichment analysis showed the first five signaling pathways. (**E**) The most important biological process enriched is the positive regulation of cell migration, which displays the differentially expressed gene network, the core part of which is composed of APP, PTGS2, IL1B, IL6, and TNF.

The gene chip detected the gene expression trend in platelets. Under the action of justicidin D, the expression of 2392 genes were up-regulated, and the expression of 3268 genes were down-regulated (Fig. 9A). Comparing these significant expression genes with the 48 common targets in the network analysis, 21 genes were screened (Fig. 9B), and enrichment analysis was performed on these 21 genes (Table 6). The GO results (the top 6 items are shown in Fig. 9C) show that the BP process involves cell migration (Fig. 9E, The core regulatory part consists of APP, PTGS2, IL1B, IL6, and TNF), inflammation, cell adhesion, and the production of inflammatory factors. CC involves the receptor complex and membrane components, while MF involves the binding of enzyme bodies and cytokine receptors. KEGG enriches multiple signaling pathways including human cytomegalovirus infection, PI3K-Akt signaling pathway, pathways in cancer, human papillomavirus infection, and human cytomegalovirus infection (Fig. 9D). This indicates that justicidin D also affects the signaling pathways related to infection in the anti-thrombotic process, which is consistent with the anti-inflammatory activity of Justicia.

**Table 6:** The GO and KEGG enrichment analysis of 21 validated genes (top 5).

Term	Category	Description	LogP	Symbols
GO:0,030,335	GO Biological Processes	Positive regulation of cell migration	− 16.978	APP, F3, IL1B, IL6, KDR, MMP9, PRKCA, PTGS2, RAC1, CXCL12, SELP, TERT, TNF, TLR2, SYK
GO:0,150,076	GO Biological Processes	Neuroinflammatory response	− 13.229	APP,IL1B,IL6,MMP9,PTGS2,TLR2,TNF,GBA,TERT,KDR,GSK3B
GO:0,045,785	GO Biological Processes	Positive regulation of cell adhesion	− 12.771	GSK3B, IL1B, IL6, KDR, PRKCA, RAC1, CXCL12, SELP, SYK, TNF, PTGS2, MMP9, APP, F3, GBA, TLR2, TERT, ABCB1
GO:0,032,757	GO Biological Processes	Positive regulation of interleukin-8 production	− 11.553	F3, IL1B, IL6, SYK, TLR2, TNF, APP, GBA, PTGS2, KDR, PRKCA, RAC1, GSK3B, MMP9, SELP
GO:0,050,900	GO Biological Processes	Leukocyte migration	− 10.3134	APP, GBA, IL6, MMP9, RAC1, CXCL12, SELP, SYK, TNF, KDR
GO:0,043,235	GO Cellular Components	Receptor complex	− 4.394	APP, IL6, KDR, SYK, TLR2
GO:0,019,898	GO Cellular Components	Extrinsic component of membrane	− 2.846	GBA, RAC1, SYK
GO:0,098,552	GO Cellular Components	Side of membrane	− 6.697	F3, ABCB1, RAC1, CXCL12, SELP, SYK, TNF, IL6, APP, IL1B
GO:0,062,023	GO Cellular Components	Collagen-containing extracellular matrix	− 2.432	F3, MMP9, CXCL12
GO:0,045,121	GO Cellular Components	Membrane raft	− 5.457	APP, LTB4R, KDR, TLR2, TNF, TERT, SELP
GO:0,005,126	GO Molecular Functions	Cytokine receptor binding	− 5.851	IL1B, IL6, CXCL12, SYK, TNF
GO:0,004,672	GO Molecular Functions	Protein kinase activity	− 3.172	GSK3B, KDR, PRKCA, SYK
GO:0,002,020	GO Molecular Functions	Protease binding	− 3.908	F3, GSK3B, TNF
GO:0,005,178	GO Molecular Functions	Integrin binding	− 7.235	IL1B, KDR, PRKCA, CXCL12, SYK, APP, MMP9, ABCB1
GO:0,035,325	GO Molecular Functions	Toll-like receptor binding	− 4.385	SYK, TLR2
hsa05163	KEGG Pathway	human Cytomegalovirus infection	− 11.335	GSK3B, IL1B, IL6, PRKCA, PTGS2, RAC1, CXCL12, TNF
hsa04151	KEGG Pathway	PI3K-Akt signaling pathway	− 8.115	GSK3B, IL6, KDR, PRKCA, RAC1, SYK, TLR2
hsa05200	KEGG Pathway	Pathways in cancer	− 8.324	GSK3B, IL6, MMP9, PRKCA, PTGS2, RAC1, CXCL12, TERT, TNF, ABCB1, SYK
hsa05165	KEGG Pathway	Human papillomavirus infection	− 5.288	GSK3B, PRKCA, PTGS2, TERT, TNF, MMP9, ABCB1, APP, TUBB1
hsa05163	KEGG Pathway	Human cytomegalovirus infection	− 11.336	GSK3B, IL1B, IL6, PRKCA, PTGS2, RAC1, CXCL12, TNF

### Justicidin D can resist platelet aggregation and prevent the lethal effect of pulmonary embolism

**Figure 10:**
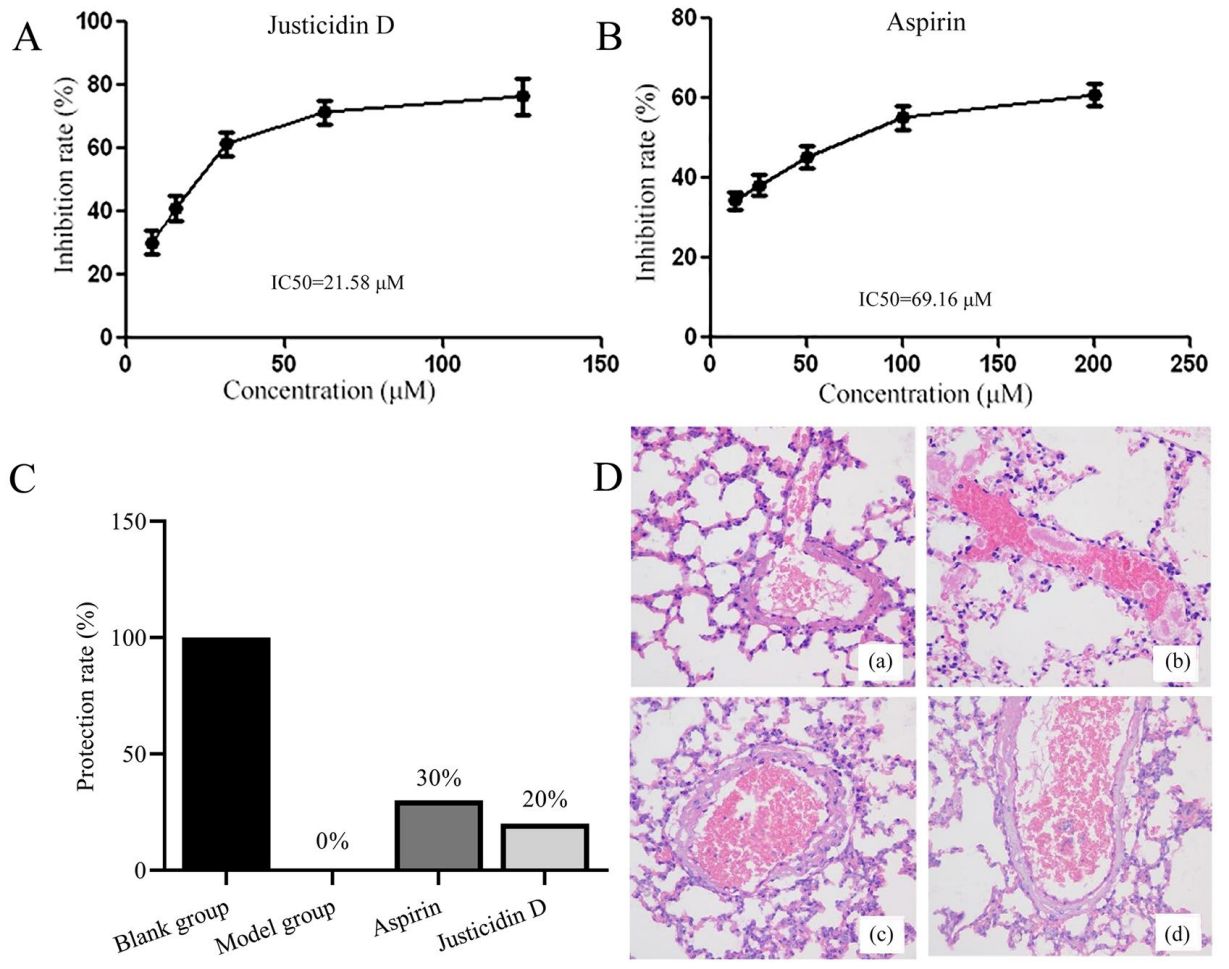
(**A**,**B**) represent the inhibitory effects of justicidin D and aspirin on platelet aggregation, respectively. (**C**) The protective effect of justicidin D and aspirin on pulmonary embolism in mice (n = 10. All survived in the control group and all died in the model group. The protection rate of justicidin D was 20%, and the protection rate of aspirin was 30%). (**D**) HE (400 ×) staining of lungs of mice with pulmonary embolism, (**a**) blank group, (**b**) model group, (**c**) justicidin D group, (**d**) aspirin group.

As shown in Fig. 10A, justicidin D has a significant inhibitory effect on ADP-induced platelet aggregation (IC50 = 21.58 μM), which is more effective than the positive control drug aspirin (a widely known anti-platelet aggregation drug, Fig. 10B, IC50 = 69.16 μM). Even more surprising is that we observed the survival of pulmonary embolism mouse models within 30 min. As shown in Fig. 10C, justicidin D, like aspirin, can effectively reduce the mortality of pulmonary embolism mice (Ten mice in each group were included in the statistical analysis. All survived in the control group and all died in the model group. The protection rate of justicidin D was 20%, and the protection rate of aspirin was 30%). By HE staining (Fig. 10D), it can be seen that the blood cells in the pulmonary blood vessels of the blank group are loose, and the blood cells in the blood vessels of the model group are tightly packed and form thrombus. Under the intervention of justicidin D, the blood cells in the blood vessels are relatively normalized, which is also consistent with the effect of aspirin. The results of animal experiments show that justicidin D has a good anti-thrombotic effect and is a potential anti-thrombotic compound. It also shows that Justicia is a Chinese herbal medicine with potential development and application value in the field of cardiovascular diseases.

## Discussion

Traditional Chinese medicine has protected the health of the Chinese people for thousands of years and has amazing curative effects. Unfortunately, the potential target of Chinese herbal medicine is difficult to determine, so far it has not yet received a high degree of international recognition. The research strategy of network pharmacology provides a feasible path for the pharmacological research of Chinese medicine. In this study, we first used network pharmacology to find important targets and potential active ingredients related to thrombosis in Justicia and predicted that multiple active ingredients would act on several proteins related to thrombosis. Then, we selected the active ingredients for experiments to verify the results of the network analysis.

In the analysis of core components, quercetin, apigenin, luteolin, cynaroside, scopoletin, palmitic acid, cleistanthin B, diphyllin, and justicidin D may be the active components with anti-thrombotic effect. Evidence has shown that quercetin can inhibit platelet activation in arterial thrombosis, and quercetin derivatives have become inhibitors of thrombosis^41,42^. As a flavonoid, apigenin has similar activity to quercetin and can inhibit platelet adhesion and thrombosis with aspirin^43^. Luteolin, as a flavonoid in Ginkgo biloba extract, is a human thrombin inhibitor, which is widely used in the prevention and treatment of thrombosis and cardiovascular disease^44^. Cynaroside, a derivative of luteolin, can reduce oxidant-induced cardiomyocyte apoptosis^45^. Scopoletin, a drug candidate for angiogenesis inhibitors, works by interrupting the autophosphorylation of VEGF receptor 2 (VEGFR2) and downstream signaling pathways, and can prevent steatosis and lower blood sugar^46^. The ratio of oleic acid to palmitic acid in the diet can determine the concentration of postprandial thrombosis and fibrinolytic factors in men^47^. There is no direct evidence that cleistanthin B and diphyllin are related to thrombosis, but they have progressive effects in inducing cell apoptosis and improving obesity^48,49^. Unfortunately, quercetin, apigenin, luteolin, cynaroside, scopoletin, palmitic acid, cleistanthin B, and diphyllin are not unique compounds in Justicia, but it is undeniable that they contribute to the anti-thrombotic effect of Justicia.

Justicidin-, a unique lignan ingredient in Justicia, has been reported to have biological activity, and Justicidin D (also called neojusticin A, Fig. 8B) is one of them. Justicidin A, a well-defined arylnaphthalide lignan, has shown anti-cancer activity and can exert neuroprotective effects by inhibiting the hyperphosphorylation of the protein tau and regulating the activities of GSK-3β and AMPK^50^. In addition, similar to Justicidin A, Justicidin B can selectively inhibit T helper 2 (Th2) cell responses in concanavalin A-activated spleen cells and polarized Th2 cells, thereby alleviating airway inflammation and bronchoconstriction^51^. Justicidin C, also called neojusticin B, has been reported to directly bind to integrin α_IIb_β_3_ to inhibit platelet aggregation^52^. Therefore, based on the results of network analysis and combined with literature reports, justicidin D was used as a key focus in this work for experimental research.

F2, MMP9, CXCL12, MET, RAC1, PDE5A, and ABCB1 are genes regulated by justicidin D identified in this work. Among them, F2, MMP9, CXCL12, and MET are also the seed nodes in the network cluster analysis and have been shown that they have a multi-dimensional connection with thrombosis. What is more, the activation and diffusion of RAC1 under high arteries significantly reduces collagen adhesion and thrombus formation^53,54^. Consistent with this, the gene chip detection in this work also showed that RAC1 was significantly high expression under the action of justicidin D (Fig. 10B). Under the intervention of justicidin D, the expression of PDE5A was up-regulated and the expression of ABCB1 was down-regulated. PDE5A can mediate and protect myocardial hypertrophy caused by hypertension and has a regulatory effect in the blood system^55^. ABCB1 participates in the transport of drugs in the blood and mediates the drug resistance of tumors^56^. In short, justicidin D inhibits thrombosis by regulating F2, MMP9, CXCL12, MET, RAC1, PDE5A, and ABCB1.

**Figure 11:**
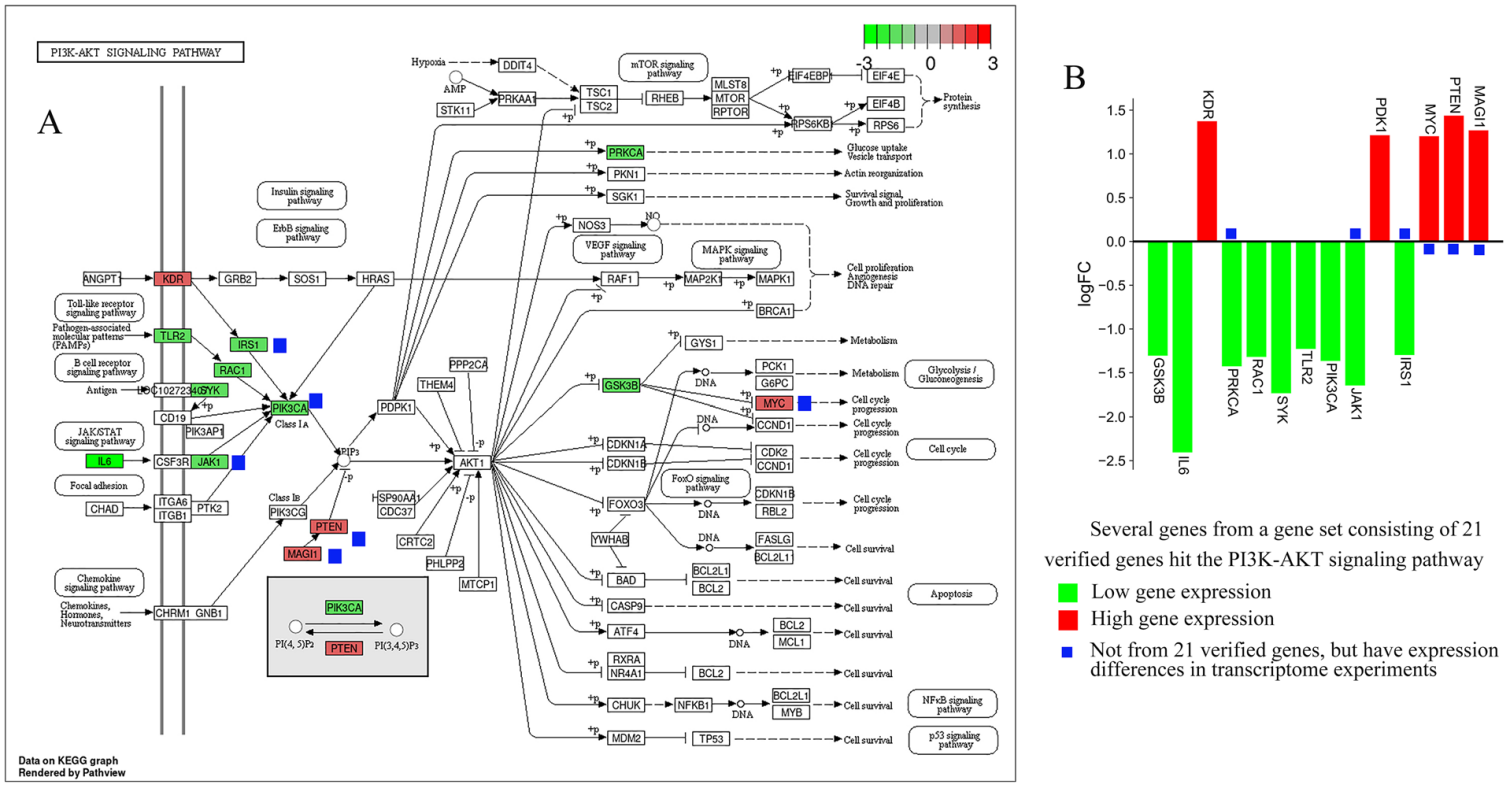
(**A**) Pathway mapping of the PI3K-Akt pathway. Green indicates down-regulation and red indicates up-regulation. (**B**) The expression of related genes in the PI3K-Akt pathway detected by the gene chip.

In addition to infection-related signaling pathways, the PI3K-Akt signaling pathway is also enriched by common targets (Supplementary Table S3) and 9 hub targets (Fig. 7B). More importantly, the differential genes regulated by justicidin D are also enriched in the PI3K-Akt signaling pathway. The differential genes involved include GSK3B, IL6, KDR, PRKCA, RAC1, SYK, TLR2, PIK3CA, JAK1, PDK1, IRS1, MYC, PTEN, MAGI1 (Fig. 11A,B). As a key node in the PI3K/Akt signaling process, the expression of PIK3CA was found to be significantly down-regulated. However, what is puzzling is that Akt1, another key node, has not been detected with significant expression changes, and the relatively low throughput of gene chip detection may explain this phenomenon. However, we also doubt whether AKT1 is still at the core in the process of thrombosis. The expanded conjecture is that the anti-thrombotic effect of justicidin D will not be mediated by AKT1. It is more likely that there is a gene downstream of PIK3CA that mediates this effect. This conjecture needs to be confirmed by in-depth experiments, but it is undeniable that PI3K-Akt signaling pathway plays a vital role in the anti-thrombotic process. Importantly, the PI3K-Akt signaling pathway is also a pathway where the predicted targets of the compounds in Justicia are commonly enriched. In-depth understanding is that the compounds in Justicia, including justicidin D, have anti-thrombotic effects through the PI3K-Akt signaling pathway.

## Conclusion

In the present study, a network pharmacology combined with experimental research analysis strategy was established to reveal the anti-thrombotic mechanism of Justicia compounds. The study found the anti-thrombotic targets, clusters, biological processes, and pathways of Justicia and confirmed that the core compounds in Justicia have antiplatelet aggregation effect and can reduce the mortality of mice with pulmonary embolism through experiments. At the same time, gene chip detection also confirmed that the characteristic components in Justicia can regulate the expression of thrombosis related genes.

## Supplementary Information

Supplementary Information.

## Data Availability

The datasets used and/or analysed during the current study were available from the corresponding author on reasonable request. The main supporting data can be found in the supplementary material of the article.
