# Supplementary material for: Reveals of candidate active ingredients in Justicia and its anti-thrombotic action of mechanism based on network pharmacology approach and experimental validation
Source: Sci Rep. 2021 Aug 25;11:17187. doi: 10.1038/s41598-021-96683-z (PMC8387432; doi:10.1038/s41598-021-96683-z)
Supplement: Supplementary file 1 — Supplementary Information. [file 41598_2021_96683_MOESM1_ESM.zip › Supplementary material/Figure S1-The 1H-NMR of Justicidin D.doc]

Figure S1 The 1H-NMR of Justicidin D
